# Response to the letter by Dr. Felix H. Blankenstein regarding “Orthodontic appliances and their diagnostic impact to brain MRI”

**DOI:** 10.1007/s00784-025-06599-5

**Published:** 2025-10-14

**Authors:** Lisa Latzko, Anna Schmit, Bernhard Glodny, Astrid E Grams, Christoph Birkl, Adriano G Crismani

**Affiliations:** 1 Department of Dental and Oral Medicine and Cranio-Maxillofacial and Oral Surgery, Medical University of Innsbruck, Innsbruck, Austria; 2 Department of Radiology, Medical University of Innsbruck, Anichstraße 35, Innsbruck, 6020 Austria; 3 Neuroimaging Core Facility, Medical University of Innsbruck, Innsbruck, Austria; 4 Department of Radiology, Medical University of Innsbruck, Anichstraße 35, Innsbruck, 6020 Austria; 5 Neuroimaging Core Facility, Medical University of Innsbruck, Innsbruck, Austria

**Keywords:** Brain MRI artifacts, Diagnostic quality, Magnetic Resonance Imaging, Orthodontic Appliances, Susceptibility-weighted imaging

## Abstract

**Objective:**

To evaluate the diagnostic impact of fixed orthodontic appliances on brain MRI under clinically realistic conditions, and to clarify the scope and focus of our Technical Note in response to commentary

**Materials and Methods:**

Common orthodontic appliances, including brackets, archwires, molar bands, and transpalatal arches, were assessed in vivo during brain MRI. The analysis was designed as a Technical Note, with emphasis on empirical diagnostic imaging rather than detailed metallurgical characterization

**Results:**

For most orthodontic configurations, brain MRI images remained interpretable across standard sequences. Severe artifacts were observed primarily in the presence of a transpalatal arch, especially in SWI and DWI sequences. These findings provide actionable insights for radiologists and orthodontists

**Conclusion:**

While theoretical alloy-based compatibility assessments remain valuable, empirical MRI data are indispensable in clinical practice, where detailed material information is often unavailable. Our study confirms that standard orthodontic appliances usually allow interpretable brain MRI, with specific exceptions

**Clinical Relevance:**

The study offers practical guidance for imaging patients with fixed orthodontic appliances. It highlights the need for interdisciplinary collaboration to establish standardized classifications of MRI-compatible products, building on both clinical imaging data and metallurgical analyses.

(*Clin Oral Investigations*, doi:10.1007/s00784-025-06275-8)

We thank Dr. Felix H. Blankenstein for his detailed and collegial commentary on our recently published Technical Note, and we appreciate the opportunity to respond.

His remarks highlight important materials science aspects - particularly the variability of stainless-steel alloys and the role of cold deformation in martensitic transformation - which we fully acknowledge.

However, we would like to take this opportunity to clarify the scope and intent of our work. Our publication was deliberately structured as a Technical Note, not as a comprehensive metallurgical analysis. The purpose was to quantify the actual diagnostic impact of commonly used orthodontic appliances on brain MRI, under clinically realistic conditions.

A key motivation for our study - and one we wish to emphasize - is precisely the fact that in daily clinical practice, detailed material data, and thus information on the magnetizability, are often unavailable. Either because manufacturers do not disclose specific alloy compositions (as current regulations permit), or because patients themselves are unaware of the exact materials present in their appliances. This practical reality means that purely theoretical or alloy-based compatibility assessments, while valuable, cannot replace empirical imaging data collected in vivo.

In that sense, our study does not attempt to generalize across all stainless steels, nor to make universal claims. Rather, it addresses the very real diagnostic question faced by radiologists and orthodontists: “Can we expect interpretable brain MRI images in the presence of standard appliances?” Our results indicate that for most configurations -brackets, archwires, molar bands - the answer is yes. Severe artifacts were observed only in the presence of a transpalatal arch, most notably in SWI and DWI sequences, providing clinically meaningful and actionable information.

We fully support Dr. Blankenstein’s suggestion to develop standardized classifications of MRI-compatible orthodontic products - such as an “Innsbruck list” - but we reiterate that such efforts must be based on interdisciplinary studies. Our study can serve as a foundation for this, particularly when paired with detailed alloy data in future work.

In conclusion, while we welcome the additional metallurgical context provided in Dr. Blankenstein’s letter, we stand by the clinical relevance, technical soundness, and focused scope of our Technical Note. We hope this exchange encourages further collaboration across disciplines, ultimately improving MRI diagnostics for patients with fixed orthodontic appliances.

Sincerely,

On behalf of the authors,

Lisa Latzko.

## Data Availability

No datasets were generated or analysed during the current study.

